# Valorization of a Local Italian Pear (*Pyrus communis* L. cv. ‘Petrucina’)

**DOI:** 10.3390/foods13101528

**Published:** 2024-05-14

**Authors:** Alessandro Frontini, Carmine Negro, Rita Accogli, Francesco Minonne, Andrea Luvisi, Luigi De Bellis

**Affiliations:** 1 Department of Biological and Environmental Sciences and Technologies (DiSTeBA), Salento University, Via Prov.le Lecce-Monteroni, 73100 Lecce, Italy; alessandro.frontini@unisalento.it (A.F.); rita.accogli@unisalento.it (R.A.); andrea.luvisi@unisalento.it (A.L.); luigi.debellis@unisalento.it (L.D.B.); 2 Comitato Esecutivo Parco “Costa Otrranto-Leuca e Bosco di Tricase”, 73039 Tricase, Italy; minonfranc@gmail.com; 3 National Biodiversity Future Center, 90133 Palermo, Italy

**Keywords:** fruit quality, antioxidants, biodiversity, local germplasm, functional food, VOCs

## Abstract

In recent decades, the food production chain has undergone transformations that have profoundly affected the way food is supplied, causing changes in the quality of the final products. Moreover, biodiversity is seriously threatened worldwide, and the valorization of local germplasm is a priority goal for most sectorial policies in Europe and elsewhere. Southern Italy and the Mediterranean basin present a vast heritage of fruit tree cultivars that is gradually being lost. Through this work, we aim to valorize a well-adapted local pear cultivar named Petrucina from the Salento area (southeastern Italy, Apulia region), which has never been studied before in detail. With this aim, the nutritional and nutraceutical features of pear flesh were characterized and compared with a reference pear cultivar that is widespread and well-known in Europe (cv. ‘Conference’). Petrucina fruits have shown a peculiar aromatic compound profile, and a content of up to 398.3, 30.9, and 4.7 mg/100 g FW of malic acid, citric acid, and ascorbic acid, respectively, much higher than that of Conference fruits. Additionally, Petrucina flesh presents a more than triple total phenolic content and an antioxidant activity more than double that of Conference, making Petrucina a true functional food that deserves wide appreciation.

## 1. Introduction

The Italian peninsula is considered an important hub of plant biodiversity, partly because of its geomorphologic diversity and its latitude extension. About 10,000 plant species have been recorded in Italy [1], presenting levels of species, habitat, and endemism in Europe [2,3]. The industrial revolution led to the emergence of specialized cropping systems, often based on the use of genetically uniform plants in monoculture systems to achieve high and consistent yields over time and to enable mechanization. Despite the great economic benefits brought by this trend, the past 150 years have seen a severe reduction in the number of plant species, increasing genetic erosion, and increasing vulnerability of the ecosystems [4]. Recently, the awareness of these issues has led to greater attention being paid to biodiversity conservation, and community policies and grants are also moving in this direction [5]. The valorization of typical genetic resources is part of this process. A local variety is defined as a variable population that has adapted to the environmental conditions of a growing area (thus tolerant to specific biotic and abiotic stresses, and therefore sufficiently productive), that has not been the subject of formal breeding, and whose products have been appreciated locally so as to receive a specific name. Over time, the local variety becomes part of the knowledge and habits of a population that continues its cultivation, although the yield is lower than that of widespread commercial cultivars [6]. However, there is growing consensus that native/local fruits and vegetables are important contributors to a varied and functional diet [7,8]. Moreover, there is a great interest in searching for foods with high contents of specific functional compounds, and there are several examples of local fruit, such as apples, that show better nutritional and nutraceutical properties in comparison with modern commercial cultivars [9,10]. Works have been published on local pear varieties from various regions of Italy [11,12,13], but the bibliography is scarce regarding pears particular to Salento. This subregion is a peninsula located at the extreme southeast of the country that has undergone partial agricultural modernization so that it currently represents an important local germplasm reservoir. Oral tradition reports the presence of a cultivar widespread in the provinces of Lecce, Brindisi, and Taranto called Petrucina, sometime also reported as the ‘Piticina’ or ‘San Pietro’ pear [14]. Little is known about it, and this name has never been mentioned as a synonym or ancestor of patented cultivars or characterized landraces. However, it is known that, throughout Salento, several farmers or garden owners claim to have pear trees called Petrucina. This fruit is part of the local tradition, and it is known for its initial astringency and high sweetness after full ripeness. For these reasons, it is commonly used for jam production after a period of post-harvest storage to reduce astringency. It is mentioned in local cookbooks [15] and in volumes on local flora [16]. In 2021, it was included in the list of the Italian Traditional Agri-food Products (PAT) [17], reporting that the fruits are small in size but particularly sugary, with white grainy flesh and a green/yellow peel with shades of red at full ripening, flowering in the first half of March, and reaching fruit maturation in the first half of July [17]. However, nobody has ever characterized it from a biochemical point of view. Thus, the aim of this work is to analyze this pear cultivar for its nutritional and nutraceutical features, and to promote its cultivation in an area of southern Italy unsuitable for the cultivation of commercial pear varieties.

## 2. Materials and Methods

### 2.1. Plant Material

**Figure 1:**
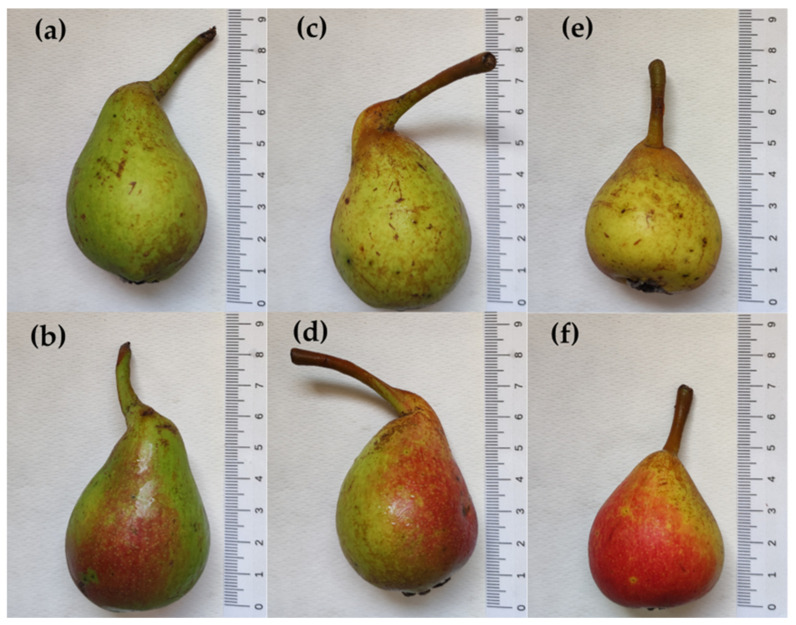
Petrucina pears. The images in the same column represent opposite sides of the same fruit (with the red-peeled side in the lower row). From left to right: t0 (**a**,**b**), t7 (**c**,**d**), t14 (**e**,**f**).

**Figure 2:**
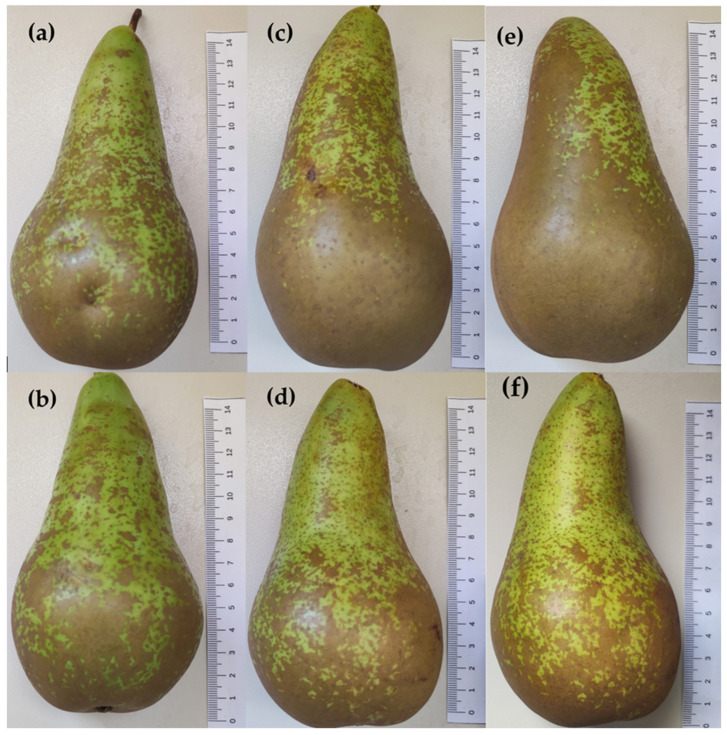
Conference pears. The images in the same column represent opposite sides of the same fruit. From left to right: t0 (**a**,**b**), t7 (**c**,**d**), t14 (**e**,**f**).

Local Petrucina pears (Figure 1) were harvested on 12 July 2023 from private parcels located in rural contexts in the municipalities of Campi and Surbo (Lecce Province, Italy). The fruits had a homogeneous appearance and three pears from each locality were analyzed the day after (t0). Then, other pears were left in the dark at 26 °C and 60% RH, and analyzed in triplicate after 7 (t7) and 14 days (t14). The cv. ‘Conference’ (Figure 2) was chosen as the reference, being the primary cultivar at the European level (EU28, before Brexit) [18]. Fruits were purchased from a local market because no commercial pear varieties are grown in the region due to the hot climate and low summer rainfall. In this case, the analyses were performed within 24 h (t0) of purchase and after 7 (t7) and 14 days (t14), respectively.

### 2.2. Morphological Parameters and Fruit Quality Analyses

For each fruit, length and diameter were measured with a dial caliper, and fresh weight was measured with an analytical balance (B3001-S, Mettler Toledo, Columbus, OH, USA). Firmness was measured with a force gauge penetrometer (FM200, PCE Instruments Ltd., Southampton, UK) with a 6 mm metallic tip. The dry weight (DW) of the flesh was determined at 105 °C until constant weight, using a thermo-ventilated oven. The following equation was used for calculation:

DW (%) = (W1 × 100)/W2(1)

where W1 is the weight after drying and W2 is the weight of the original sample.

Each fruit was peeled, cut into smaller pieces, and the central core removed. A part of the aliquoted flesh was stored at −80 °C until analyses. Another part was immediately homogenized employing a HOMEX 6 instrument (Bioreba, Reinach, Switzerland); total soluble solids were measured with a digital refractometer (PDR-70, XS instruments, Carpi, MO, Italy), while total titratable acidity (TTA) was measured by titration of 10 mL of juice.

Sugars (d-glucose, d-fructose, and sucrose) and organic acids (l-malic acid, citric acid, and ascorbic acid) were quantified with commercial enzymatic kits (K-SUFRG, K-LMAL, K-CITR, K-ASCO, respectively, Megazyme Int., Wicklow, Ireland), reading absorbances with a JASCO V-550 UV/VIS spectrophotometer (Mary’s Ct, Easton, MD, USA).

### 2.3. Antioxidant Compounds Extraction and Analyses

Flesh stored at −80 °C was ground under liquid nitrogen and 500 mg of the powder was immediately mixed with 5 mL of the extraction solvent (60% methanol, 39% water, 1% formic acid) in 15 mL tubes. These were left for 20 min in a thermostatic ultrasonic bath (DU-45, Argo Lab, Carpi, Modena, Italy) set to 40 kHz and 40 °C. Then, the samples were centrifuged, the liquid phase was collected, and the pellet discarded. These extracts were used to determine antioxidant activity, total phenolic content, condensed tannin content, and HPLC analyses.

The total phenolic content (TPC) was determined using the spectrophotometric Folin–Ciocalteu method, as indicated in Negro et al. [19]. Absorbance was measured with a JASCO V-550 UV/VIS spectrophotometer at 765 nm, and data were expressed as gallic acid equivalent (GAE) per mg/g dry weight (DW). Antioxidant activity was determined in vitro by evaluation of the free radical scavenging activity using DPPH assay (2,2-diphenyl-1-picrylhydrazyl (DPPH)) [20], ABTS assay (2,2′-azinobi-(3-ethylbenzothiazoline-6-sulfonic acid (ABTS)) [20], and FRAP assay (ferric reducing antioxidant power) [21]. Calculations of antioxidant values were realized after drawing a standard curve with different concentrations of 6-hydroxy-2,5,7,8-tetramethylchroman-2-carboxylic acid (Trolox) [21]. Inhibition of free radical DPPH was expressed as EC50 (μg of fresh weight of pear flesh in methanol 60%). Condensed tannin content was determined using the acidified vanillin method [20]. ABTS and FRAP results were reported as µmol of Trolox equivalents (TE) per 100 g of fresh weight of pear flesh.

### 2.4. HPLC/DAD/TOF

Phenolic compounds were identified by an Agilent 1200 liquid chromatography system (Agilent Technologies, Palo Alto, CA, USA) equipped with a standard autosampler, following the protocol reported in Negro et al. [20]. The HPLC column was an Agilent extended C18 (1.8 μm, 2.1 × 50 mm). Separation was carried out at 40 °C with a gradient elution program at a flowrate of 0.5 mL/min. The mobile phases consisted of water plus 0.1% formic acid (A) and acetonitrile (B). The following multistep linear gradient was applied: 0 min, 5% B; 13 min, 25% B; 19 min, 40% B. The injection volume in the HPLC system was 5 μL. The HPLC system was coupled to a diode array detector (DAD, 1260 Infinity, Agilent Technologies) reading at 280 nm and an Agilent 6320 TOF mass spectrometer equipped with a dual electrospray ionization (ESI) interface (Agilent Technologies) operating in negative ion mode. Detection was carried out within a mass range of 50–1700 *m*/*z*, and the mass spectrometer conditions were as follows: capillary voltage 3.0 kV in negative mode; nitrogen as the nebulizer and desolvation gas; drying gas temperature 300 °C; drying gas flow 12 L/min; nebulizing gas pressure 40 psig; finally, the source temperature was 120 °C. Mass Hunter software version B.07.00 (Agilent Technologies) was used to process the mass data of the molecular ions. Accurate measurements of the mass corresponding to each total ionic current (TIC) peak were obtained with a pump (Agilent G1310B) introducing a low flow (20 μL/min) of a calibration solution containing internal reference masses at *m*/*z* 112.9856, 301.9981, 601.9790, and 1033.9881, and using a dual nebulizer ESI source in negative ion mode [20].

### 2.5. Gas Chromatography/Mass-Spectrometry

Volatile organic compounds (VOCs) emitted by whole pears were analyzed by the SPME method, essentially as previously reported [22]. Fruits were sealed in a 780 mL capacity glass jar, whose lid was modified to allow the insertion of the syringe, maintaining airtightness. After sealing, the SPME syringe was inserted, and the fiber (50/30 µm divinylbenzene/carboxen/polydimethylsiloxane, Supelco/Merck KGaA, Darmstadt, Germany), which was previously conditioned for 5 min at 235 °C in the gas chromatograph injector, was exposed overnight at room temperature to absorb the volatile compounds.

Subsequently, the fiber was inserted into the injector port of a gas chromatography with a mass spectrometry detector (Agilent 7890B coupled with MS single quadrupole Agilent 5977A), and the desorption of the volatile compounds was performed at 235 °C for 4 min. At this point, the chromatographic run was started by employing an Agilent HP-5 ms column (30 m × 0.25 mm, 0.25 µm); the temperature was raised from 60 °C to 230 °C with a constant increase of 3 °C/min, with a helium (purity > 99.999%) constant flow of 1.0 mL/min. Compounds were identified by library search and analytical standards. The mass spectrum of an unknown compound was searched in a data-processing system [23]. Substances with a score above 800, both for identity and purity, were putatively identified after comparing the detected compound with the one in the NIST Computational Chemistry Comparison and Benchmark database [23]. Retention index (RI) was obtained, as reported by Zhao et al. [24], in comparison with the retention times of a series of C8–C20 alkanes separated under the GC-MS conditions mentioned above, and the following formula was applied:

(2) $$\mathrm{RI}=100\times n+\frac{100\;\left(t_{a-}t_{n}\right)}{t_{n+1}-t_{n}}$$

where *t_a_* is the retention time of the unknown peak *a*; *t_n_* is the retention time of *n*-alkane *C_n_*; *t_n_*_+1_ is the retention time of *n*-alkane *C_n_*_+1_; *n* = carbon number of the alkane that elutes before the unknown peak *a*.

### 2.6. Sensory Test

A sensory test, as described by Min Allah et al. [25] with some modifications, was conducted by 5 University of Salento employees, 3 women and 2 men. Tasters were chosen randomly and without any kind of tasting technique background; it was intentionally decided to obtain feedback comparable to that of ordinary consumers. The test was conducted on both Petrucina and Conference pears. Fruits were peeled, cut into equal form/size slices, placed in two unlabeled dishes, and administered randomly so that the panelists would hopefully not immediately recognize the source. They were asked to taste and assign a score (0, 1, 2, 3, 4, 5, corresponding, respectively, to absent, very low, low, medium, high, very high) to the intensity of five different sensory perceptions (sweetness, acidity, astringency, aroma, crunchiness). Finally, they were asked to assign a score to the general pleasantness (from 0 to 5). The results were reported as the average of the scores assigned by each taster.

### 2.7. Statistics

All data were reported as the mean ± standard deviation (SD), with at least three replications for each sample. Statistical evaluation was conducted by a one-way analysis of variance (ANOVA) and Tukey’s test for honestly significant differences (HSD) to discriminate among the mean values. All statistical analyses were performed using the R software (version 4.0.3, R Core Team [26]).

## 3. Results

### 3.1. Morphological Parameters

The measurements confirmed the smaller size of the Petrucina pear compared with the Conference pear (about half the length and about five times less weight) (Table 1).

### 3.2. Fruit Quality Analyses

Just after harvest, the Petrucina pear presented a firmness of 45.1 N and a juice Brix degree of 14.5, while a Conference pear with a similar firmness had a Brix degree of 10.2 (Table 2). After 7 and 14 days in controlled conditions, Petrucina juice reached 17.0 and 19.5 ° Brix, respectively, while the firmness of the fruit decreased to 26.3 and 16.7, respectively. The Conference pear, after 14 days, reached, at most, 15.5 ° Brix and 9.0 N firmness.

**Figure 3:**
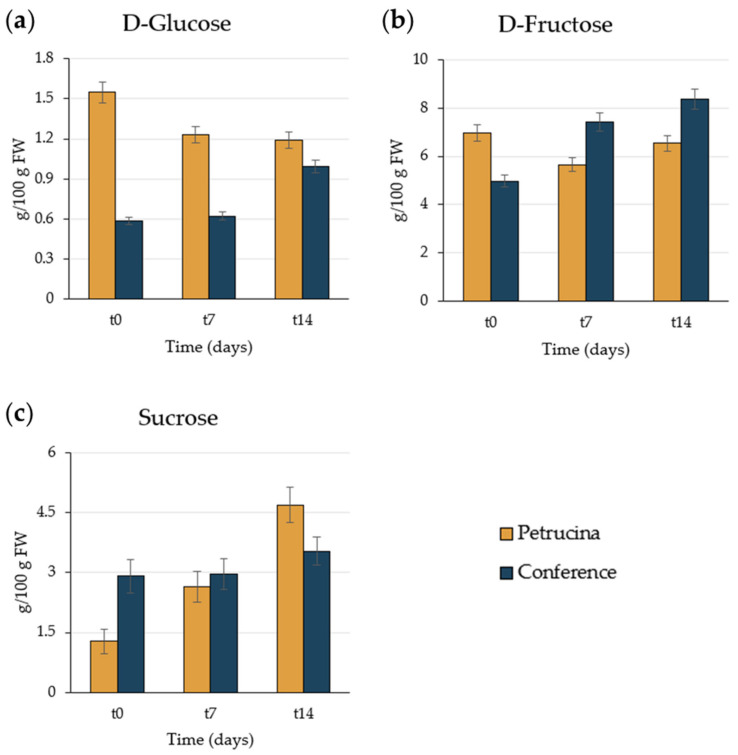
Sugar content (g) in 100 g of fresh weight of flesh: (**a**) D-glucose, (**b**) D-fructose, (**c**) sucrose. The statistically significant difference between each cultivar and each time was assessed by one-way ANOVA followed by Tukey’s post hoc test (HSD).

Regarding sugar development, different pears presented distinct trends; in fact, while in the Petrucina pear the D-glucose content decreased as days went by, in the Conference pear it increased (Figure 3a). D-fructose content, however, remained approximately stable for the Petrucina pear but increased as the Conference pear ripened (Figure 3b). For both kinds of fruit, the sucrose content increased over time, but in Petrucina the increase results were higher, reaching 4.70 g/100 g FW at t14 (Figure 3c), more than three times the content recorded just after harvest (1.34 g/100 g FW).

**Figure 4:**
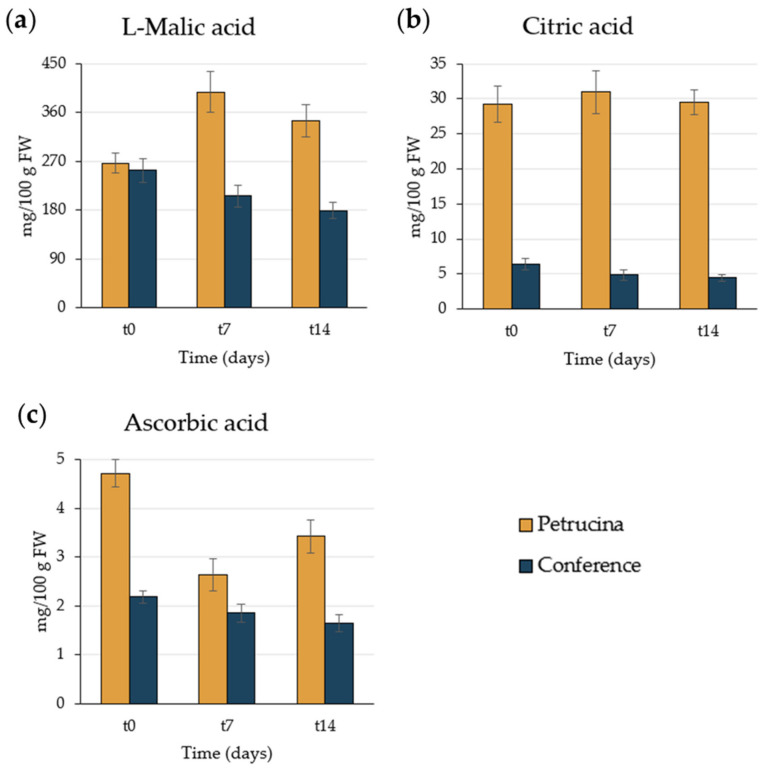
Organic acid content (mg) in 100 g of fresh weight of flesh: (**a**) L-malic acid, (**b**) citric acid, (**c**) ascorbic acid. The statistically significant difference between each cultivar and each time was assessed by one-way ANOVA followed by Tukey’s post hoc test (HSD).

In the case of organic acid content, the difference among cultivars was more prominent (Figure 4). L-malic acid had an increase at t7 and then remained stable in Petrucina but decreased gradually for Conference. Concerning ascorbic acid, its values started from 4.7 mg/100 g FW at t0, diminished to 2.6 mg/100 g FW at t7, and then changed to 3.4 mg/100 g FW for Petrucina, while dropping slightly for Conference, starting from approximately 2 mg/100 g FW. The difference between cultivars was significant at t0 and t14, with content doubled in Petrucina. Really interesting was the difference in citric acid content between the two cultivars, approximately six times greater in Petrucina than Conference at all stages.

### 3.3. Antioxidant Compounds Extraction and Analyses

**Figure 5:**
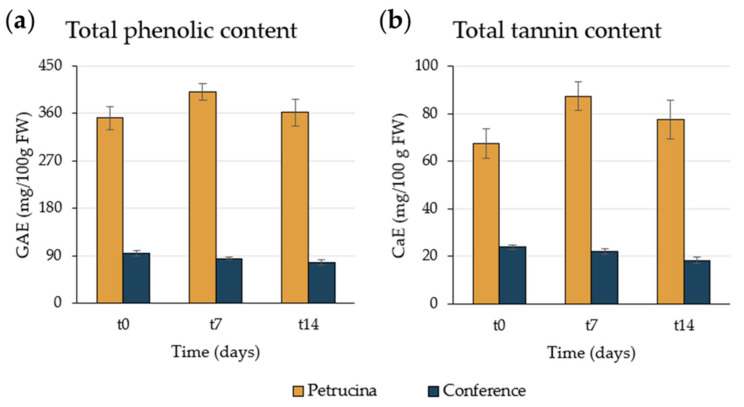
Content of (**a**) total phenolic compounds (TPC, expressed as gallic acid equivalents, GAE) and (**b**) condensed tannin content (expressed as catechin equivalents, CaE) expressed as mg/g of fresh weight of flesh. The statistically significant difference between each cultivar and each time was assessed by one-way ANOVA followed by Tukey’s post hoc test (HSD).

Petrucina fruit presented a higher content of total phenolic compounds and condensed tannins than Conference fruit, statistically significant at all stages (Figure 5). The Conference pear had less than a third of total phenolic compounds compared with the Petrucina pear and one-third of condensed tannins on average. In the Petrucina pear, the highest level of both phenolic compounds and tannins was registered at t7.

Consistently, the results from each antioxidant activity test showed that it was considerably higher in the case of Petrucina flesh without significant changes from t0 to t14 (lower EC50 values for DPPH assay and higher values for ABTS and FRAP assays); instead, for Conference flesh, the lower antioxidant activity decreased from t0 to t14 (Table 3).

#### HPLC/DAD/TOF

**Figure 6:**
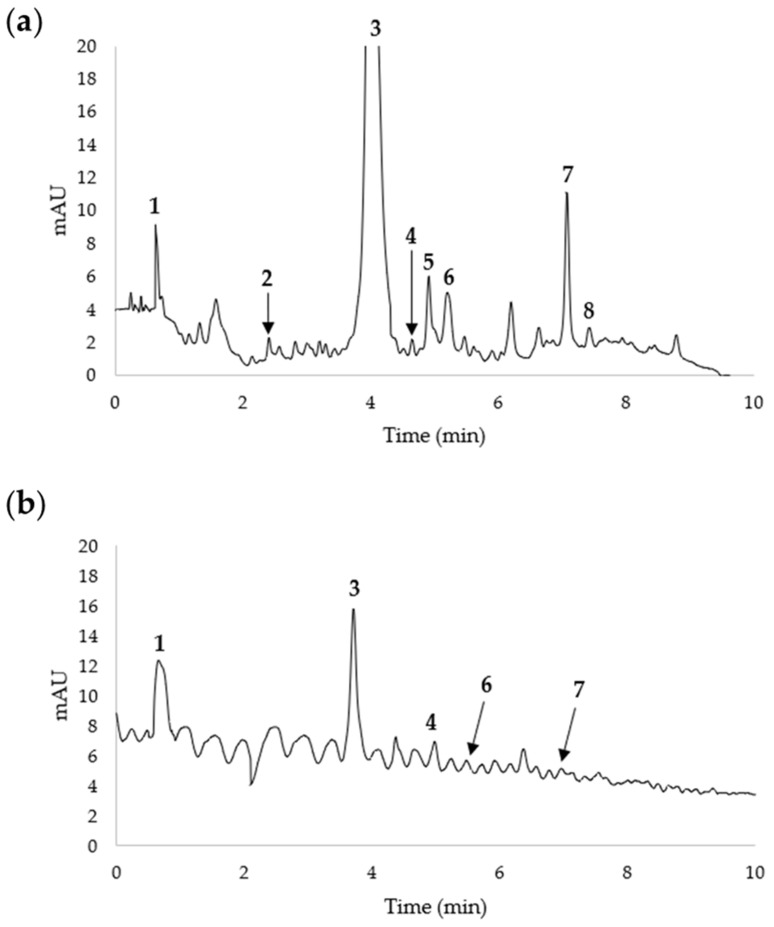
HPLC/DAD chromatogram of pear flesh of (**a**) Petrucina and (**b**) Conference pear at t14. The peak numbers refer to the compounds listed in Table 4.

Figure 6 shows the analyses conducted at t14 when fruits were consumed. The analyses pointed out the presence of eight main compounds in the flesh of the Petrucina pear, of which only five were detected in the flesh of the Conference pear (Figure 6, Table 4). Both chromatograms show that the main compound was caffeoylquinic acid (3), followed by feruloyl quinic acid (7) in Petrucina, while quinic acid was the main compound in the flesh of the Conference pear.

The results indicated that the flesh of Petrucina contained a higher level of secondary metabolites in comparison with the flesh of Conference, suggesting that the local pear of Salento—adapted to a hot and dry summer climate—produced additional specific secondary metabolites. The three compounds detected only in Petrucina—phenolic compound hydroxybenzoic acid, quinic acid derivate coumaroylquinic acid, and flavan-3-ol gallocatechin-3-O-glucose—are known as inducers of resistance and scavengers against oxidative stress in plants; together with caffeoylquinic acid—the more abundant secondary metabolite in Petrucina flesh—they are considered beneficial for human health.

### 3.4. GC/MS

**Figure 7:**
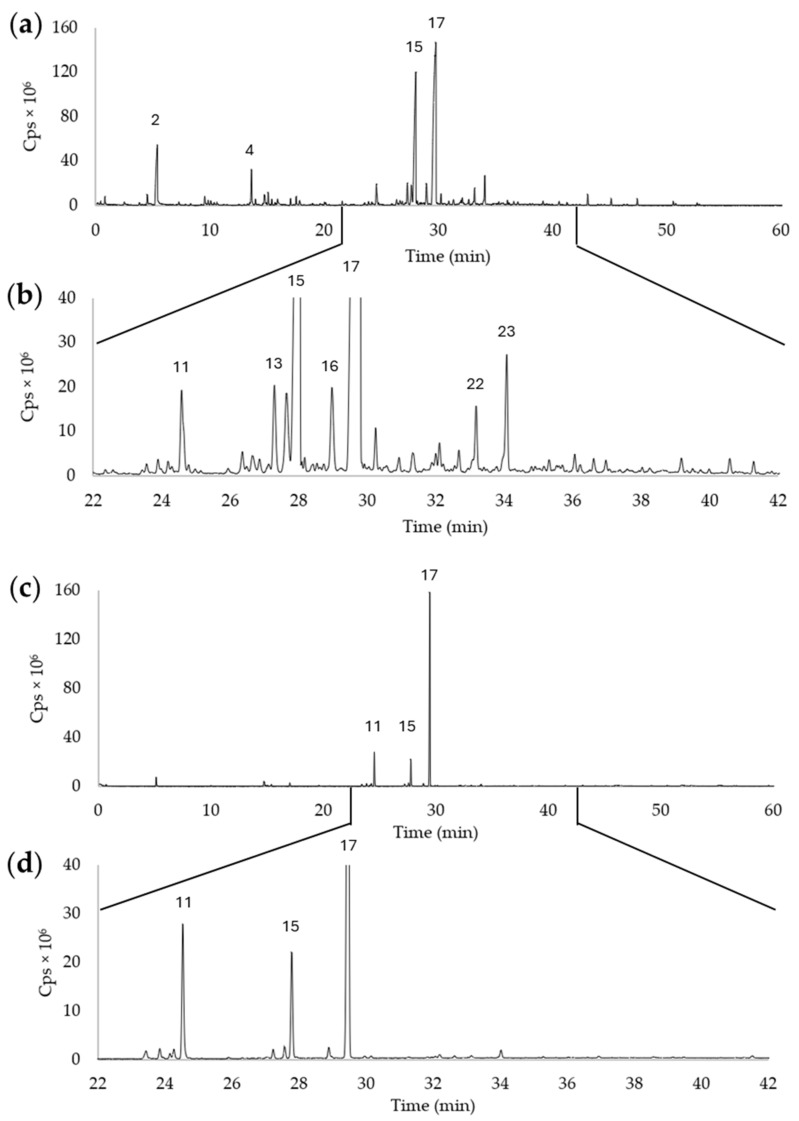
Chromatograms of volatile compounds emitted by whole pears. (**a**,**b**) Petrucina; (**c**,**d**) Conference; (**b**,**d**) are restricted sections of the chromatogram (**a**) and (**c**), respectively. The peak numbers refer to the compounds listed in Table 4.

The analyses of the volatile organic compounds (VOCs) emitted by the pears (shown in Figure 7) were realized from fruits at t7, because this stage should represent a plausible flavor profile at the time of product selection/purchase by the consumer. Therefore, Figure 7 is representative of the results of GC/MS analyses, which were essentially similar at the three stages. Even in this case, we detected more volatile chemical compounds by analyzing fruits from Petrucina than from Conference (23 compared with 13, Table 5). The two pears had nine compounds in common. In both cases, the main compound was α-farnesene (Figure 7), with a relative peak area of 40.9% and 65.9% for Petrucina and Conference, respectively. It is a linear sesquiterpene derived from the cytosolic mevalonic acid pathway and is found as two different isomers: E,E-α-farnesene and Z,E-α-farnesene. Other sesquiterpenes were detected: copaene (only in Conference), α-himachalene, (+)-ledene (only in Petrucina), and α-bergamotene and γ-bisabolene (in both cultivars). Only in Petrucina, two isomers of bisabolene were found, with the remarkable percentages of 1.3% and 2.6%. The second more abundant compound was ethyl (E,Z)-2,4-decadienoate for Petrucina and methyl (E,Z)-2,4-decadienoate for Conference. Compared with these, most other volatile compounds emitted by the pears were fatty acid esters. Figure 7 demonstrates the complexity of the VOCs produced by the Petrucina pear.

### 3.5. Sensory Test

**Figure 8:**
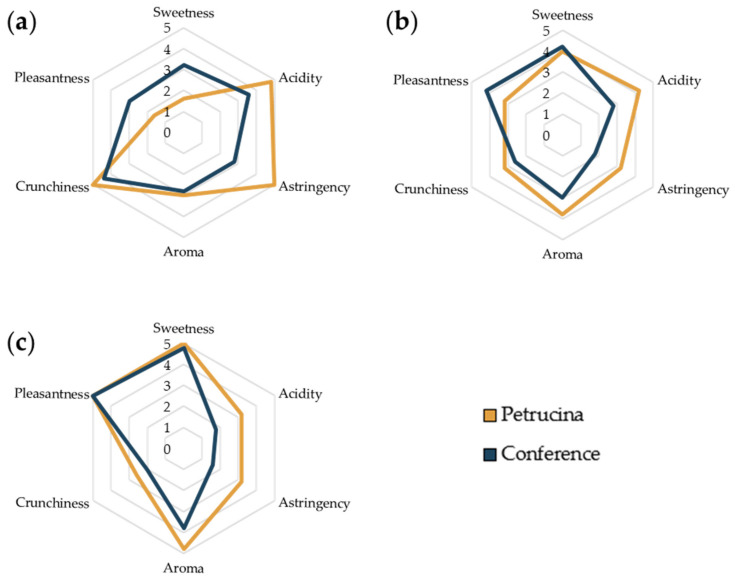
Radar chart on sensory test on Petrucina and Conference flesh at (**a**) t0, (**b**) t7, and (**c**) t14. Scores from 0 to 5 correspond to the intensity of the perception (absent, very low, low, medium, high, very high).

The sensory test results highlighted that the Petrucina pear results were unpleasant just after harvest (score of 1.6/5), with most intensity towards the perceptions of acidity and astringency (Figure 8a); in fact, astringency and acidity were perceived as very high by five out of five and four out of five tasters, respectively. Then, at t7 and t14, the scores assigned to these two perceptions progressively decreased, and those assigned to sweetness and pleasantness increased, reaching the values awarded to the Conference pear. It was interesting that the perception of aroma (which was greater than in the Conference pear) reached an average score of 4.8/5 and 3.8/5 for Petrucina and Conference, respectively (Figure 8c), and for crunchiness (average score 2.6 for Petrucina and 2.0 for Conference).

## 4. Discussion

Morphological analysis has confirmed that the Petrucina pear is a small fruit, thus, in principle, poorly suited for the market (despite the presence of the red-shaded side of the peel appearing attractive), but biochemical analysis has shown that this pear possesses a number of interesting characteristics. At harvest, Petrucina presents a similar flesh firmness to Conference, and a higher total soluble solids content but, interestingly, it was unpleasant in the sensory test (two tasters considered Petrucina’s pleasantness as very low and three considered it as low, while a medium pleasantness was assigned to Conference, Figure 8a) maybe due to the high levels of tannins and citric acid combined with a low level of total sugars (Figure 3, Figure 4 and Figure 5). At t7, the sweet perception increased but some astringency remained, influencing the general pleasantness (rated as medium by four out of five tasters, Figure 8b). At t14, the flesh of Petrucina resulted as sweet and pleasant, with an average score of 5 for both, corresponding to very high perceptions, and for Conference (Figure 8c). These observations were consistent with the total soluble solids content measurements. In fact, 14.5 °Brix and 19.5 °Brix were recorded for Petrucina at t0 and t14, respectively, while for Conference it moved from 10.2 °Brix to 15.5 °Brix. The result was a sharp increase in the taster’s perception of sweetness and appreciation as days went by after harvest for Petrucina. The increase in Brix degree was probably due to the increased sucrose content as d-glucose decreased, and the d-fructose remained approximately stable during the ripening phase. It cannot be ruled out that these changes resulted from the synthesis of sucrose from d-glucose and d-fructose, as reported by Lee et al. [32], who reported a positive correlation between the activity of the enzyme sucrose phosphate synthase and cell wall invertase. Regarding organic acids, in Petrucina, L-malic acid unexpectedly increased at t7, and then slightly decreased, yet citric acid (whose content was significantly higher than in Conference) did not show significant changes. Ascorbic acid had a significant reduction at t7 and then increased again; in general, it remained higher than in Conference. Cascia and colleagues [33] observed the post-harvest ascorbic acid content in fruits of three pear cultivars, noticing a general reduction except for a slight increase after two weeks, as in our results. Other authors [34] examined the behavior of three pear cultivars (two Asian cultivars, KS9 and KS13, and one European cultivar, Shahmiveh) during post-harvest storage at 1 °C, showing in KS9 an accumulation of malic acid after one month and then a decline after two and four months. This trend could be comparable to that observed for Petrucina, even if, in our case, the modifications occurred more quickly (maybe due to the different storage temperature). At the same time, the authors observed a progressive reduction of malic acid in the remaining two cultivars (like in Conference) [34]. Lindo-García et al. [35] suggested that the pear ripening pattern is cultivar-dependent, which affects the time and the trend of maturation after harvest. The observations on Petrucina and KS9 were consistent with the evidence of Akhavan and Wrolstad [36] regarding the organic acid trends during shelf-life storage in the Bartlett pear harvested at an immature stage. They observed an initial accumulation of malic acid and then a reduction, as shown for Petrucina (Figure 4). These results are promising for the high content of ascorbic acid or vitamin C (whose importance in human health is well documented [37]), while the presence of high amounts of phenolic compounds makes it more interesting due to their high antioxidant activity, remaining at high levels even at t7 and t14 (Table 3). Though it is broadly reported that phenolic compounds tend to decrease during ripening and storage [38] (as occurred in Conference, Figure 5a), different behaviors could be observed, depending on genotype and environmental conditions. In fact, if some authors reported a progressive decline in phenolic content during long storage at 0.5 °C [39], others observed a discontinuous course during shelf life at room temperature [40], as in this case. Wang and colleagues [41] reported an increase in total phenolic content during the first week after harvest and then a rapid reduction. They suggested that the synthesis of phenolic compounds can be promoted and regulated by gene coding for enzymes involved in phenylpropanoid metabolism, maybe activated in connection with the positive effect of phenolic compounds in preventing or reducing the effects of stresses caused by both biotic and abiotic factors. Focusing on qualitative analysis of phenols, caffeoylquinic acid corresponds to the main peak after the HPLC/MS analysis on flesh of both cultivars, as reported by Kolniak-Ostek [27]. Caffeoylquinic acid is also called chlorogenic acid and is part of the hydroxycinnamic acid family. It presents a considerable antibacterial activity [42] and possesses several beneficial effects for human health, like hepatoprotective, cardioprotective, neuroprotective, and general anti-inflammatory action [43]. It is one of the most important phenolic acids assimilable with diet [44]. Commisso et al. [45] stated that chlorogenic acid is a phenolic compound scarcely accumulated in market available cultivars and, instead, it is abundant in local pear varieties. The compounds found in the flesh of the Petrucina pear have also been found in the flesh of the Radana pear by other authors [27], except for quinic acid, found only in the peel, and hydroxybenzoic acid, not found. In addition, Wang and colleagues [29] detected each compound found in this work (Table 4) in the pulp of different cultivars of Australian pears, excluding quinic acid. Hudina et al. [46] reported the results of HPLC analyses on the flesh of the Conference pear and the phenolic profile was similar to that observed in our results (differing only for arbutin and feruloyl quinic acid) [46]. Also, in apple, some authors found a higher number of phenolic compounds and greater antioxidant activity in local cultivars than in those easily available on the market [47]. It is well known that polyphenols influence the taste of food and, in general, its sensory perception [48], thus the complexity of the Petrucina phenolic profile can provide feedback in terms of consumer appreciation. In addition, the flavor (determined by the volatile compounds) is also crucial for consumer purchase choice regarding fruit [49]. The VOCs found in higher concentrations are α-farnesene and ethyl (E,Z)-2,4-decadienoate for both pears, while, in the case of Petrucina, hexyl acetate also showed a discrete relative peak area (9.8%) (Table 5). α-farnesene, which provides a woody green herbaceous odor with a lavender and citric background [31], has been indicated as a major volatile of intact pears by other authors [50]. At the same time, α-farnesene plays an important role in plant–insect interactions [51]. The esters of 2,4-decadienoate are molecules responsible for providing some of the most distinctive odors of the aroma in several pear varieties [52]. The pure ethyl (E,Z)-2,4-decadienoate smell green, waxy, pear, apple, sweet, fruity, and tropical [31]. Hexyl acetate was found as one of the main VOCs of *Pyrus communis* [53] and it provides a fruity aroma (with apple, pear, and banana nuances) with green and fresh notes [31]. In Petrucina, its relative content in term of peak total area was three time greater than in Conference (Table 4). In addition, two isomers of γ-bisabolene were detected in Petrucina. This molecule provides a balsamic, citric, terpenes, fruity aroma [31,54] and it has been proven that it has anticancer potential [55]. Other interesting compounds identified exclusively in Petrucina were ethyl hexanoate, ethyl 2,4-hexadienoate, ethyl octanoate, ethyl-(E)-2-octenoate, ethyl palmitate, and methyl palmitate. These compounds contribute to creating a complex aromatic profile in the Petrucina pear, although present only in traces. The odor type of ethyl hexanoate, ethyl 2,4-hexadienoate, and ethyl-(E)-2-octenoate is fruity, with a tendency to pineapple, anise, and pear, respectively [31]. Ethyl octanoate, ethyl palmitate, and methyl palmitate, instead, contribute to providing a waxy and fatty aroma [31]. Ethyl hexanoate and ethyl octanoate were found by GC analyses in different cultivars of *Pyrus communis* grown in Brazil [56]. Qin and colleagues [57] analyzed the volatile compounds of 33 different cultivars of *Pyrus ussuriensis* grown in China: interestingly, they detected the presence of ethyl 2,4-hexadienoate in only 3 cultivars. In this work, the presence of ethyl and methyl palmitate was not reported; these compounds were emitted, instead, by Egyptian pears [58].

## 5. Conclusions

Considering the interest toward local germplasm recovery and promotion, this work provides information useful for the valorization of a local pear cultivar, typical of the Salento area, southeastern Italy. The results collected support the view that local fruit cultivars, compared with modern cultivars, possess higher nutritional and nutraceutical characteristics. For this reason, the Petrucina pear is a candidate worthy of belonging to the functional food category because of its high content of assimilable substances such as nutrients, vitamins, and antioxidant compounds and other secondary metabolites whose intake is closely related to reducing the incidence of various diseases and increasing life expectancy. Moreover, the intriguing aromatic profile provides further opportunity for this product to be accepted by consumers [58], as long as the time between harvest and consumption is correctly managed. In fact, consumer appreciation depends on the level of ripeness of the fruits (being very low at an early stage of ripening). On the other hand, the possibility of maintaining high levels of antioxidant activity during ripening makes post-harvest storage possible without compromising the beneficial characteristics and may allow the Petrucina pear to be appreciated as a typical product. Lastly, the perspective of obtaining some fruit products from traditional crops (adapted for many years to the local environment and, therefore, requiring low input) to be sold in the local market, thus avoiding long-distance transportation, could be a strategy for providing healthy food in compliance with environmental issues, according to the principles of the ‘farm to fork strategy’ promoted by the European Union.

## Figures and Tables

**Table 1:** Morphological parameters of fruits.

Cultivar	Length (cm)	Diameter (cm)	Fresh Weight (g)
Petrucina	5.3 ± 1.3 ^b^	4.3 ± 0.7 ^b^	39.2 ± 5.1 ^b^
Conference	12.0 ± 0.6 ^a^	6.8 ± 0.4 ^a^	261.3 ± 33.5 ^a^

Values are the mean ± standard deviation (*n* = 9). The statistically significant difference between each cultivar and each time was assessed by one-way ANOVA followed by Tukey’s post hoc test (HSD). Means in a column with different letters differ at *p* < 0.05.

**Table 2:** Ripening indexes in fresh fruits.

Cultivar	Stage	Dry Weight (%)	Total Soluble Solids (°Brix)	Total Titratable Acidity (g Malic Acid/L)	Firmness (N)
Petrucina	t0	15.1 ± 1.1 ^ab^	14.5 ± 0.2 ^d^	1.8 ± 0.1 ^a^	45.1 ± 6.6 ^a^
	t7	18.2 ± 1.5 ^a^	17.0 ± 0.3 ^b^	1.6 ± 0.1 ^b^	26.3 ± 4.4 ^bc^
	t14	18.8 ± 1.9 ^a^	19.5 ± 0.6 ^a^	1.2 ± 0.1 ^c^	16.7 ± 2.3 ^cd^
Conference	t0	11.2 ± 0.9 ^c^	10.2 ± 0.1 ^f^	1.5 ± 0.1 ^b^	44.3 ± 4.9 ^a^
	t7	14.1 ± 1.2 ^bc^	13.2 ± 0.2 ^e^	1.2 ± 0.2 ^c^	32.1 ± 4.1 ^b^
	t14	15.6 ± 1.5 ^ab^	15.5 ± 0.4 ^c^	0.9 ± 0.1 ^d^	9.0 ± 2.0 ^d^

Values are the mean ± standard deviation (*n* = 3). The statistically significant difference between each cultivar and each time was assessed by one-way ANOVA followed by Tukey’s post hoc test (HSD). Means in a column with different letters differ at *p* < 0.05.

**Table 3:** Antioxidant activity assays: DPPH, ABTS, FRAP.

Variety		DPPH (EC50, µg FW)	ABTS (µmol TE/100 g FW)	FRAP (µmol TE/100 g FW)
Petrucina	t0	135.4 ± 5.4 ^d^	177.0 ± 7.6 ^a^	231.1 ± 5.3 ^a^
	t7	141.4 ± 4.2 ^d^	197.4 ± 6.5 ^a^	243.3 ± 8.7 ^a^
	t14	156.1 ± 3.1 ^d^	197.2 ± 9.4 ^a^	233.9 ± 4.8 ^a^
Conference	t0	238.1 ± 12.1 ^c^	128.4 ± 7.3 ^b^	174.6 ± 8.8 ^b^
	t7	282.6 ± 8.5 ^b^	79.2 ± 5.8 ^c^	98.9 ± 5.3 ^c^
	t14	340.0 ± 13.6 ^a^	66.8 ± 2.1 ^c^	74.4 ± 12.7 ^d^

Values are the mean ± standard deviation (*n* = 3). The statistically significant difference between each cultivar and each time was assessed by one-way ANOVA followed by Tukey’s post hoc test (HSD). Means in a column with different letters differ at *p* < 0.05.

**Table 4:** Compounds [M-H]^−^ identified by HPLC/DAD/TOF in pear flesh. In the last two columns, + or − indicates the presence or the absence in the flesh of Petrucina (Pet) and Conference (Con) pear.

N	RT	Name	*m*/*z* Exp	*m*/*z* Calc	Δ ppm	[M-H]^−^	Ref.	Pet	Con
1	0.589	Quinic acid	191.0213	191.0197	5.49	C_7_H_11_O_6_	[27,28,29]	+	+
2	2.598	Hydroxybenzoic acid	137.0244	137.0244	0.59	C_7_H_5_O_3_	[28]	+	−
3	4.006	Caffeoylquinic acid	353.0886	353.0878	−1.35	C_16_H_17_O_9_	[27,28,29,30]	+	+
4	4.535	Procyanidin dimer	577.1348	577.1351	0.97	C_30_H_25_O_12_	[27,28,29,30]	+	+
5	4.987	Coumaroylquinic acid	337.0926	337.0929	2.57	C_16_H_17_O_8_	[27,28,29]	+	−
6	5.31	(+)-Catechin	289.0716	289.0718	0.35	C_15_H_13_O_6_	[26,27,28,29]	+	+
7	7.14	Feruloyl quinic acid	367.1029	367.1035	−1.19	C_17_H_20_O_9_	[26,27,28,29]	+	+
8	7.423	Gallocatechin-3-O-glucose	481.0962	481.0988	0.22	C_21_H_21_O_13_	[28]	+	−

**Table 5:** Volatile organic compounds (VOCs) emitted by whole fruits. Numbers in the Petrucina and Conference columns indicate the relative area (%) of the peaks.

No.	RI	Compound Name	Peak Area (%)	
			Petrucina	Conference
1	996	Ethyl hexanoate	0.7	
2	1011	Hexyl acetate	9.5	2.8
3	1093	Ethyl 2,4-hexadienoate	0.8	
4	1156	1-octene, 3-(methoxymethoxy)-	3.0	
5	1187	Butyl hexanoate		2.6
6	1196	Ethyl octanoate	1.0	
7	1227	Hexyl 2-methyl butyrate	0.5	1.1
8	1246	Ethyl-(E)-2-octenoate	0.6	
9	1376	Copaene		0.9
10	1380	Cyclohexanebutanol, 2-methyl-3-oxo-, cis-		0.8
11	1394	Methyl (E,Z)-2,4-decadienoate ^1^	2.2	10.5
12	1428	Unknown	0.5	
13	1457	Ethyl (E,Z)-2,4-decadienoate isomer 1 ^2^	2.2	0.7
14	1463	Unknown	2.6	0.9
15	1471	Ethyl (E,Z)-2,4-decadienoate isomer 2 ^2^	21.4	8.1
16	1498	α-bergamotene	2.2	1.0
17	1510	α-farnesene ^3^	40.9	65.9
18	1517	α-himachalene	0.8	
19	1534	(+)-ledene	0.5	
20	1549	Unknown	0.5	
21	1557	Unknown	0.4	
22	1562	γ-bisabolene isomer 1 ^4^	1.3	0.8
23	1595	γ-bisabolene isomer 2 ^4^	2.6	
24	1837	Unknown	0.8	
25	1928	Methyl palmitate	0.5	
26	1990	Ethyl palmitate	0.5	
27	2140	Oleic acid		1.1

^1^ The odor description of methyl (E,Z)-2,4-decadienoate (2,4-decadienoic acid, methyl ester) is fruity (pear, lemon), waxy [31]; ^2^ the odor description of ethyl (E,Z)-2,4-decadienoate (2,4-decadienoic acid, ethyl ester) is green, waxy, pear, tropical [31]; ^3^ the odor description of α-farnesene is woody, green, herbal, citrus [31]; ^4^ the odor description of γ-bisabolene is fruity, balsamic, woody, citrus, terpenes [31].

## Data Availability

The original contributions presented in the study are included in the article, further inquiries can be directed to the corresponding author.

## References

[1] Bartolucci F., Peruzzi L., Galasso G., Albano A., Alessandrini A., Ardenghi N.M.G., Astuti G., Bacchetta G., Ballelli S., Banfi E. (2018). An Updated Checklist of the Vascular Flora Native to Italy. Plant Biosyst..

[2] Galasso G., Conti F., Peruzzi L., Ardenghi N.M.G., Banfi E., Celesti-Grapow L., Albano A., Alessandrini A., Bacchetta G., Ballelli S. (2018). An Updated Checklist of the Vascular Flora Alien to Italy. Plant Biosyst..

[3] Bartolucci F., Galasso G., Peruzzi L., Conti F. (2021). Report 2020 on Plant Biodiversity in Italy: Native and Alien Vascular Flora. Nat. Hist. Sci..

[4] Zimmerer K.S., de Haan S., Jones A.D., Creed-Kanashiro H., Tello M., Carrasco M., Meza K., Plasencia Amaya F., Cruz-Garcia G.S., Tubbeh R. (2019). The Biodiversity of Food and Agriculture (Agrobiodiversity) in the Anthropocene: Research Advances and Conceptual Framework. Anthropocene.

[5] Labianca M. Towards the New Common Agricultural Policy for Biodiversity: Custodian Farmers for Sustainable Agricultural Practices in the Apulia Region (South of Italy). Belgeo.

[6] Gigli M., Ministero delle Politiche Agricole e Forestali (2013). Linee Guida per La Conservazione e La Caratterizzazione Della Biodiversità Vegetale Di Interesse per l’agricoltura.

[7] Cogill B. (2014). Contributions of Indigenous Vegetables and Fruits to Dietary Diversity and Quality. Proceedings of the XXIX International Horticultural Congress on Horticulture: Sustaining Lives, Livelihoods and Landscapes (IHC2014): International Symposium on Promoting the Future of Indigenous Vegetables Worldwide.

[8] Zimmerer K.S., De Haan S. (2017). Agrobiodiversity and a Sustainable Food Future. Nat. Plants.

[9] Berni R., Cantini C., Guarnieri M., Nepi M., Hausman J.-F., Guerriero G., Romi M., Cai G. (2019). Nutraceutical Characteristics of Ancient *Malus x domestica* Borkh. Fruits Recovered across Siena in Tuscany. Medicines.

[10] Sut S., Zengin G., Maggi F., Malagoli M., Dall’Acqua S. (2019). Triterpene Acid and Phenolics from Ancient Apples of Friuli Venezia Giulia as Nutraceutical Ingredients: LC-MS Study and in Vitro Activities. Molecules.

[11] Draga S., Palumbo F., Miracolo Barbagiovanni I., Pati F., Barcaccia G. (2023). Management of Genetic Erosion: The (Successful) Case Study of the Pear (*Pyrus communis* L.) Germplasm of the Lazio Region (Italy). Front. Plant Sci..

[12] Bergonzoni L., Alessandri S., Domenichini C., Dondini L., Caracciolo G., Pietrella M., Baruzzi G., Tartarini S. (2023). Characterization of Red-Fleshed Pear Accessions from Emilia-Romagna Region. Sci. Hortic..

[13] Ferradini N., Lancioni H., Torricelli R., Russi L., Ragione I.D., Cardinali I., Marconi G., Gramaccia M., Concezzi L., Achilli A. (2017). Characterization and Phylogenetic Analysis of Ancient Italian Landraces of Pear. Front. Plant Sci..

[14] Livraghi Verdesca Zain G. (1994). Tre Santi e Una Campagna. Culti Magico-Religiosi Nel Salento Di Fine Ottocento.

[15] Cordella M.F. (2015). La Cucina Salentina: Fra i Piatti Della Tradizione. L’Idomeneo.

[16] Savino V.N., Palasciano M., Lipari E., Mazzeo A., Pacucci C., Todisco M.C., Losciale P., Gaeta L., Minonne F., Biscotti N., Lillo A. (2018). Atlante Dei Frutti Antichi Di Puglia.

[17] Regione Puglia (2021). Bollettino Ufficiale Della Regione Puglia-n. 160 Del 23-12-2021.

[18] Musacchi S., Iglesias I., Neri D. (2021). Training Systems and Sustainable Orchard Management for European Pear (*Pyrus communis* L.) in the Mediterranean Area: A Review. Agronomy.

[19] Negro C., Tommasi L., Miceli A. (2003). Phenolic Compounds and Antioxidant Activity from Red Grape Marc Extracts. Bioresour. Technol..

[20] Negro C., Aprile A., Luvisi A., De Bellis L., Miceli A. (2021). Antioxidant Activity and Polyphenols Characterization of Four Monovarietal Grape Pomaces from Salento (Apulia, Italy). Antioxidants.

[21] Xiao F., Xu T., Lu B., Liu R. (2020). Guidelines for Antioxidant Assays for Food Components. Food Front..

[22] Frontini A., De Bellis L., Luvisi A., Blando F., Allah S.M., Dimita R., Mininni C., Accogli R., Negro C. (2022). The Green Leaf Volatile (Z)-3-Hexenyl Acetate Is Differently Emitted by Two Varieties of *Tulbaghia violacea* Plants Routinely and after Wounding. Plants.

[23] NIST (National Institute of Standards and Technology) (2022). Computational Chemistry Comparison and Benchmark Database NIST Standard Reference Database Number 101.

[24] Zhao Y.Z., Li Z.G., Tian W.L., Fang X.M., Su S.K., Peng W.J. (2016). Differential Volatile Organic Compounds in Royal Jelly Associated with Different Nectar Plants. J. Integr. Agric..

[25] Min Allah S., Dimita R., Negro C., Luvisi A., Gadaleta A., Mininni C., De Bellis L. (2023). Quality Evaluation of Mustard Microgreens Grown on Peat and Jute Substrate. Horticulturae.

[26] (2020). R Core Team R: A Language and Environment for Statistical Computing. https://www.R-project.org/.

[27] Kolniak-Ostek J., Oszmiański J. (2015). Characterization of Phenolic Compounds in Different Anatomical Pear (*Pyrus communis* L.) Parts by Ultra-Performance Liquid Chromatography Photodiode Detector-Quadrupole/Time of Flight-Mass Spectrometry (UPLC-PDA-Q/TOF-MS). Int. J. Mass Spectrom..

[28] Simirgiotis M.J., Quispe C., Bórquez J., Areche C., Sepúlveda B. (2016). Fast Detection of Phenolic Compounds in Extracts of Easter Pears (*Pyrus communis*) from the Atacama Desert by Ultrahigh-Performance Liquid Chromatography and Mass Spectrometry (UHPLC-Q/Orbitrap/MS/MS). Molecules.

[29] Wang Z., Barrow C.J., Dunshea F.R., Suleria H.A.R. (2021). A Comparative Investigation on Phenolic Composition, Characterization and Antioxidant Potentials of Five Different Australian Grown Pear Varieties. Antioxidants.

[30] Teixeira J.C., Ribeiro C., Simôes R., Alegria M.J., Mateus N., de Freitas V., Pérez-Gregorio R., Soares S. (2023). Characterization of the Effect of a Novel Production Technique for ‘Not from Concentrate’ Pear and Apple Juices on the Composition of Phenolic Compounds. Plants.

[31] The Good Scents Company Providing Information for the Flavor, Fragrance, Food and Cosmetic Industries. http://www.thegoodscentscompany.com/.

[32] Lee B.R., Cho J.H., Wi S.G., Yang U., Jung W.J., Lee S.H. (2021). The Sucrose-to-Hexose Ratio Is a Significant Determinant for Fruit Maturity and Is Modulated by Invertase and Sucrose Re-Synthesis during Fruit Development and Ripening in Asian Pear (*Pyrus pyrifolia* Nakai) Cultivars. Hortic. Sci. Technol.

[33] Cascia G., Bulley S.M., Punter M., Bowen J., Rassam M., Schotsmans W.C., Larrigaudière C., Johnston J.W. (2013). Investigation of Ascorbate Metabolism during Inducement of Storage Disorders in Pear. Physiol. Plant..

[34] Arzani K. (2008). Postharvest Physicochemical Changes and Properties of Asian (*Pyrus serotina* Rehd.) & European (*Pyrus communis* L.) Pear Cultivars. Hort. Environ. Biotechnol..

[35] Lindo-García V., Muñoz P., Larrigaudière C., Munné-Bosch S., Giné-Bordonaba J. (2020). Interplay between Hormones and Assimilates during Pear Development and Ripening and Its Relationship with the Fruit Postharvest Behaviour. Plant Sci..

[36] Akhavan I., Wrolstad R.E. (1980). Variation of Sugars and Acids During Ripening of Pears and in the Production and Storage of Pear Concentrate. J. Food Sci..

[37] Iqbal K., Khan A., Khattak M.M.A.K. (2004). Biological Significance of Ascorbic Acid (Vitamin C) in Human Health—A Review. Pak. J. Nutr..

[38] Gundewadi G., Reddy V.R., Bhimappa B. (2018). Physiological and Biochemical Basis of Fruit Development and Ripening—A Review. J. Hill Agric..

[39] Kaur S., Singh Gill M., Gill P.P.S., Jawandha S.K., Prem Singh N. (2023). Influence of Harvest Date on Storage Quality of Asian Pear (Pyrus Pyrifolia) Fruit. Erwerbs-Obstbau.

[40] Zhao Z., Xu G., Han Z., Li Q., Chen Y., Li D. (2013). Effect of Ozone on the Antioxidant Capacity of “Qiushui” Pear (*Pyrus pyrifolia* Nakai cv. Qiushui) during Postharvest Storage. J. Food Qual..

[41] Wang J., Lv M., He H., Jiang Y., Yang J., Ji S. (2020). Glycine Betaine Alleviated Peel Browning in Cold-Stored ‘Nanguo’ Pears during Shelf Life by Regulating Phenylpropanoid and Soluble Sugar Metabolisms. Sci. Hortic..

[42] Lou Z., Wang H., Zhu S., Ma C., Wang Z. (2011). Antibacterial Activity and Mechanism of Action of Chlorogenic Acid. J. Food Sci..

[43] Singh S.K., Thakur K., Sharma V., Saini M., Sharma D., Vishwas S., Kakoty V., Pal R.S., Chaitanya M.V.N.L., Babu M.R. (2023). Exploring the Multifaceted Potential of Chlorogenic Acid: Journey from Nutraceutical to Nanomedicine. S. Afr. J. Bot..

[44] Naveed M., Hejazi V., Abbas M., Kamboh A.A., Khan G.J., Shumzaid M., Ahmad F., Babazadeh D., FangFang X., Modarresi-Ghazani F. (2018). Chlorogenic Acid (CGA): A Pharmacological Review and Call for Further Research. Biomed. Pharmacother..

[45] Commisso M., Bianconi M., Poletti S., Negri S., Munari F., Ceoldo S., Guzzo F. (2021). Metabolomic Profiling and Antioxidant Activity of Fruits Representing Diverse Apple and Pear Cultivars. Biology.

[46] Hudina M., Stampar F. (2011). Effect of Fruit Bagging on Quality of “Conference” Pear (*Pyrus communis* L.). Eur. J. Hort. Sci..

[47] Preti R., Tarola A.M. (2021). Study of Polyphenols, Antioxidant Capacity and Minerals for the Valorisation of Ancient Apple Cultivars from Northeast Italy. Eur. Food Res. Technol..

[48] De Oliveira L.D.L., De Carvalho M.V., Melo L. (2014). Health Promoting and Sensory Properties of Phenolic Compounds in Food. Rev. Ceres..

[49] Diehl D.C., Sloan N.L., Bruhn C.M., Simonne A.H., Brecht J.K., Mitcham E.J. (2013). Exploring Produce Industry Attitudes: Relationships between Postharvest Handling, Fruit Flavor, and Consumer Purchasing. Horttechnology.

[50] Yi X.K., Liu G.F., Rana M.M., Zhu L.W., Jiang S.L., Huang Y.F., Lu W.M., Wei S. (2016). Volatile Profiling of Two Pear Genotypes with Different Potential for White Pear Aroma Improvement. Sci. Hortic..

[51] Scutareanu P., Bruin J., Posthumus M.A., Drukker B. (2003). Constitutive and Herbivore-Induced Volatiles in Pear, Alder and Hawthorn Trees. Chemoecology.

[52] Zierer B., Schieberle P., Granvogl M. (2016). Aroma-Active Compounds in Bartlett Pears and Their Changes during the Manufacturing Process of Bartlett Pear Brandy. J. Agric. Food Chem..

[53] Chen Y., Yin H., Wu X., Shi X., Qi K., Zhang S. (2018). Comparative Analysis of the Volatile Organic Compounds in Mature Fruits of 12 Occidental Pear (*Pyrus communis* L.) Cultivars. Sci. Hortic..

[54] Mahmoud E., Ramadan M., Ismail M., Fadel M., Abass M. (2022). Production of Flavors from Agro Waste of *Ocimumbasilicum* L. by Different Microorganisms Using Solid-State Fermentation. Egypt. J. Chem..

[55] Jou Y.J., Hua C.H., Lin C.S., Wang C.Y., Wan L., Lin Y.J., Huang S.H., Lin C.W. (2016). Anticancer Activity of γ-Bisabolene in Human Neuroblastoma Cells via Induction of P53-Mediated Mitochondrial Apoptosis. Molecules.

[56] Wendt L.M., Ludwig V., Thewes F.R., Soldateli F.J., Batista C.B., Fukui C.M., Gonçalves dos Santos G., Katsurayama J.M., Brackmann A., Both V. (2024). Effect of Dynamic Controlled Atmosphere on Volatile Compound Profile and Quality of Pears. Sci. Hortic..

[57] Qin G., Tao S., Cao Y., Wu J., Zhang H., Huang W., Zhang S. (2012). Evaluation of the Volatile Profile of 33 *Pyrus ussuriensis* Cultivars by HS-SPME with GC-MS. Food Chem..

[58] El-Hawary S.S., El-Tantawi M.E., Kirollos F.N., Hammam W.E. (2018). Chemical Composition, in Vitro Cytotoxic and Antimicrobial Activities of Volatile Constituents from *Pyrus communis* L. and *Malus domestica* Borkh. Fruits Cultivated in Egypt. J. Essent. Oil-Bear. Plants.

